# GPs’ attitudes towards the diagnosis and treatment of male urinary tract infections: a qualitative interview study in Ireland

**DOI:** 10.3399/bjgpopen19X101667

**Published:** 2019-10-16

**Authors:** Róisín Fallon, Karen Farrell, Genevieve Leon, Aparna Rajan, Sinead Duane, Christine FitzGerald, Marie Tierney, Akke Vellinga

**Affiliations:** 1 Research Assistant, Department of General Practice, School of Medicine, National University of Ireland, Galway, Ireland; 2 Health Research Board Primary Care, Clinical Trials Network, Ireland; 3 House officer, Grade UD41Department of General Practice, School of Medicine, National University of Ireland Galway, Galway, Ireland; 4 Post Doctoral Researcher, Health Research Board Trials Methodology Research Network, College of Medicine, Nursing & Health Sciences, National University of Ireland, Galway, Ireland; 5 Research Assistant, School of Business and Economics, National University of Ireland, Galway, Ireland; 6 Postdoctoral Researcher, Health Research Board Primary Care Clinical Trials Network, National University of Ireland, Galway, Ireland; 7 Department of General Practice, School of Medicine, National University of Ireland, Galway, Ireland; 8 Epidemiologist and Senior Lecturer, Health Research Board Primary Care Clinical Trials Network, National University of Ireland, Galway, Ireland; 9 Department of General Practice, School of Medicine, National University of Ireland, Galway, Ireland; 10 Discipline of Bacteriology, School of Medicine, National University of Ireland, Galway, Ireland

**Keywords:** male, urinary tract infection, general practice, attitudes, treatment, diagnosis

## Abstract

**Background:**

In general practice, males represent around 20% of the total number of urinary tract infection (UTI) consultations. The majority of UTI research focuses on the diagnosis and treatment of women with UTIs but there is little evidence on how male UTIs are treated.

**Aim:**

To better understand GPs’ attitudes towards the diagnosis and treatment of male UTIs. This research aimed to support future investigations to determine best practice in diagnosis and treatment of male UTI.

**Design and setting:**

A qualitative interview study was carried out with 15 GPs across Ireland.

**Method:**

A topic guide was created to ensure consistency in interviews. The interviews were audiorecorded and transcribed verbatim. Transcripts were analysed using thematic analysis.

**Results:**

Fifteen interviews with GPs were completed. Analysis indicated that GPs’ knowledge of guidelines and implementation of them varied widely when deciding a treatment plan for a male presenting with UTI symptoms. There was clear consensus that male UTIs were uncommon and complicated to diagnose. Three GPs reported never treating a male UTI, while others reported treating <5 patients in their careers. There was an assumption that sexually transmitted infections (STI) take precedence in young males when presenting with similar symptoms. The use of antimicrobial treatment guidelines varied widely, in line with the interpretation of the origin and severity of symptoms.

**Conclusion:**

Male UTIs are perceived by GPs as rare and complicated. GPs expressed that patient age, resources, and guidelines available limited their confidence in diagnosing and treating male UTIs.

## How this fits in

Little research exists on how best to diagnose and treat a male UTI. Even though UTIs are less frequently observed in males compared to females, they can often be more serious and research is needed to better understand GPs attitudes towards male UTI diagnosis and treatment. This study provides greater insight into treatment and diagnosis of male UTI symptoms in general practice, and identifies where knowledge gaps exists.

## Introduction

UTIs are one of the most common conditions seen by GPs in female patients, and the most frequent bacterial infection identified in women,^1,2^ accounting for 25% of all infections^3^ even though males still represent 20% of the total number of UTI consultations.^4^ The main body of research on UTIs has focused on females because of the much higher prevalence and incidence, however, the risk of a male developing a UTI remains. Risk increases substantially with age and the vast majority of men aged >85 years have underlying urologic and functional abnormalities that can impair normal voiding.^5–7^ The most common is benign prostatic hyperplasia, known to cause UTIs, resulting in obstruction and disorderly urine flow. Acute bacterial prostatitis (a prostate infection)^8^ and, in some cases, chronic bacterial prostatitis stem from recurrent UTIs.^9^ UTIs in males are considered uncommon, with 7.8 cases (95% confidence interval [CI] = 5.1 to 10.6) per 100 per year at risk for males aged >85 years, compared to 12.8 (95% CI = 10.4 to 15.2) per 100 per year at risk for females.^10^

UTIs in males are often viewed as complicated as they are not as common as female UTIs in primary care.^11,12^ This could be due to the development of complications causing the treatment to be prolonged and expensive.^4^ First-line treatment for males and females in Ireland is the same (nitrofurantoin or trimethoprim) but differs in the recommended duration, with males to take a 7-day course and females a 3-day course.^13^ Fluoroquinolones are the recommended second-line treatment and are considered more effective in treating those who are significantly or systemically unwell.^13^ Fluoroquinolones are often preferred over cephalosporins and nitrofurantoin for their ability to penetrate prostatic fluid.^12^ However, an observational veteran study did not favour fluoroquinolones over trimethoprim, showing that duration of treatment (7 days) was more important in relation to long-term cure.^14^ Due to the growing problem of antimicrobial resistance (AMR) worldwide, there is a concerted effort to limit the use of antimicrobials, in particular second-line or ‘reserve’ antimicrobials.^14,15^

The present study aimed to understand GPs’ attitudes towards diagnosis and treatment of male patients with UTIs, to gain a better understanding of the decision-making process for prescribing antibiotics specifically to male patients, and to support and develop better guidance in relation to antimicrobial prescribing for male UTIs.

### Method

Purposeful non-probability sampling was used to recruit GPs in this study. In June 2016, GPs who were part of the discipline’s GP network were invited for interview. The GPs selected to participate were from different geographic locations and both male and female, thus minimising bias and increasing generalisability. A semi-structured interview format was adopted to allow the flow of the interview without restricting the emergence of information. A topic guide was created based on previous literature on how male UTIs are diagnosed^16,17^ to ensure all the questions asked during the interview remained consistent. The topic guide examined factors that influence the GPs’ decision on how to diagnose and treat a male UTI; how the decision to categorise it as an (un)complicated UTI is made; which other factors are taken into account; and what, if any, medication is prescribed. Box 1 outlines the key topics discussed with the GP and the sample of guiding questions. Three pilot interviews were conducted, with two face-to-face interviews and one telephone interview, to ensure all questions were clear and no difficulties in answering the questions were reported. Interviews were conducted by two researchers (fourth year medical students; one asking the questions while the other took notes). This enhanced the reflexivity of the research as the interviewers’ medical background and knowledge would have contributed to the dialogue with the interviewees (GPs) in terms of medical terminology and clinical familiarity. An academic supervisor was present for all pilot interviews. Based on the experience the researchers had with the initial GP interviews, no new questions were added to the interview schedule, and no issues arose from the pilot interviews; all interviews were included in the final analysis. Two team members conducted face-to-face interviews in the GP’s practice between June and September 2016 with recruitment continuing until data saturation. If face-to-face interview was not possible, a telephone interview was set up and the same procedure was carried out. Participants were requested to sign a consent form before the interview commenced and complete a demographic questionnaire. Each interview was audiorecorded and transcribed verbatim, and averaged about 20 minutes. All transcripts and recordings were then encrypted and password protected, and were only accessible by members of the team.

Box 1 **GP interview guide**

**Table 1a:** 

**Section 1: Male UTI in general**
Can you describe a ‘typical’ male UTI patient?
Can male UTIs be uncomplicated?
How would you treat a suspected male UTI?
Why the chosen treatment?
**Section 2: Influencing factors in treatment decisions**
Are male patients different (in treatment of UTI)?
What other external factors influence treatment decision for UTI in males?
Is AMR a factor considered when prescribing antibiotics for male UTI?
**Section 3: Issues with male UTI**
Do you have other concerns in relation to male UTIs?
How do you approach these concerns?
Are there specific circumstances?

AMR = antimicrobial resistance UTI = urinary tract infection

### Analysis

Data saturation was based on the information as it was collected during the interviews. Once no new information emerged during the interviews, as agreed by both researchers, the interviews were stopped. The transcripts were open-coded and labelled by two independent researcher groups to identify the interesting segments related to the research objectives. After the coding process, researchers discussed the findings and developed the analytical framework which emerged from the open codes and labels. Sets of codes (themes) were identified, defined, coded, and categorised using N-Vivo (version 11). All themes were developed retrospectively. The themes were compared and findings were discussed to ensure rigour within the analysis process. Three dominant themes emerged, providing insights into the diagnosis and prescribing behaviour of the GPs in relation to male UTIs:

- GPs’ experience of diagnosing and treating male UTIs;
- GPs’ challenges in diagnosing and treating male UTIs; and
- GPs’ interactions with patients during male UTI consultation.

## Results

### Demographics

A total of 15 GPs were interviewed; 5 face-to-face and 6 telephone interviews. The 15 GPs worked in 13 different practices, with 11 in urban practices and 2 rural. The 8 females and 7 males were in practice for <10 hours per week (*n* = 2), 10–19 hours (*n* = 6), 20–29 hours (*n* = 4) or for >30 hours (*n* = 3). Their experience in general practice ranged from >15 years (*n* = 2), to <5 years (*n* = 5). When asked if they used the antibiotic prescribing guidelines on a routine basis, 8 GPs said they always did, 5 GPs often, and 2 GPs never consulted the guidelines. The guidelines most often referred to were the Health Service Executive (HSE) guidelines by 9 GPs, National Institute for Health and Care Excellence (NICE) guidelines by 2 GPs, and local lab guidelines by 1 GP; another GP confirmed using guidelines but they did not specify which.

The findings describe GPs’ experience, challenges, and interaction with diagnosing and treating suspected UTI in male patients.

### GPs’ experience of diagnosing and treating male UTIs

The majority of GPs had little experience in treating male UTIs, indicating a low frequency of male UTI consultations in their practices. Twelve of the 15 GPs spoke of male UTIs as *‘rare’* or ‘*uncommon*’, highlighting that the majority of UTI cases they consult are females or children:

*‘I would say that it is uncommon to see men with urinary tract infections. The vast majority are either women or children … So its incidence is low.*’ (Male, urban practice)

*‘What I believe about male UTIs … they’re rare.’* (Male, urban practice)

A lack of familiarity, knowledge, and experience of male UTIs was outlined and acknowledged by 9 of the 15 GPs:

*‘I suppose in general practice, you can kind of get into little areas of comfort and I suppose the treatment of male UTIs I suppose wouldn’t be one I’d be hugely comfortable with.’* (Female, rural practice)

The GPs who had experience with treating male UTIs indicated they were taken aback with a male presenting with a UTI or, in other words, GPs take note when one presents:

*‘It makes me sit up.’* (Male, urban practice)

*‘… you shouldn’t be getting a urinary tract infection … What’s wrong with you? Are you okay? You know, yeah, so it’s almost like backward.’* (Male, urban practice)

GPs report that male patients have specific expectations when receiving care from their GP. GPs suggest these patients want a definitive diagnosis, to match the symptoms they present with and a treatment that will work for them:

*‘My experience over an awful number of years is that generally, that they want the diagnosis first, that they want to say, so this is happening, and this is happening … they want the diagnosis and of course they want a plan to confirm treatment.’* (Male, urban practice)

*‘I know what this is, I’ve had it many times, I always get an antibiotic.’* (Male, urban practice)

However, diagnosing a male UTI is considered a more taxing process in comparison to a typical female UTI consultation in general practice:

*‘… you’d be looking … scratching the surface a bit … so a bit deeper.’* (Female, urban practice)

*‘… I think one’s got to look for an underlying cause.’* (Male, urban practice)

The lack of understanding of how or why the male UTI occurs is what drives this perception of UTIs being difficult to diagnose and complex. GPs question their clinical judgment when diagnosing a male UTI:

‘*So you don’t get male UTIs without a cause … So, if you have a male UTI, there is something causing it.’* (Male, urban practice)

*‘... because you have to think laterally outside the box, I think they’re all a bit complicated … with a small “c”.’* (Male, urban practice)

Nine out of the 15 GPs considered all male UTIs to be complicated:

*‘The causes of urinary symptoms in men are more likely to be something complicated.’* (Male, urban practice)

*‘I suppose I would kind of view almost all, all male UTIs as complicated … I would probably have the suspicion that they would* [be complicated] *yeah, whereas I wouldn’t with females.’* (Female, urban practice)

Only five of the participants were of the opinion that male UTIs could be uncomplicated:

*‘I don’t think they all are* [complicated]*, no I wouldn’t.’* (Female, rural practice)

### GPs’ challenges in diagnosing and treating male UTIs

GPs make clinical judgments in order to appropriately diagnose and treat patients. This is sometimes challenged within a clinical setting and results in a situation of trial and error:

*‘Hmm so it’s what you want to make sure you have the diagnosis correct and that you’re not actually, you know, making an incorrect diagnosis when you are treating them, that it’s there, that you haven’t overlooked anything and that if you are giving antibiotics that you’ve got the sensitivities right.’* (Female, urban practice)

*‘… make a clinical judgment, inevitably when you do that, inevitably you’re going to get it right from the timing most of the time.’* (Male, urban practice)

GPs are cautious in their diagnosis and treatment of male UTIs, they want to make sure they have explored all avenues, and exhausted any underlying causes as to why males have these symptoms. They are sceptical in their treatment options and want to make sure they are prescribing appropriately. GPs question their clinical judgment when diagnosing a male UTI:

*‘Whether I am missing something, whether I should be referring to some guidelines, I don’t know but I don’t think so … So I would tend to find it easier.’* (Male, urban practice)

Male UTIs are typically treated with antibiotics but there is much confusion and interpretation of guidelines:

*‘I use them to shorten illness even though I have no idea what I am treating.’* (Male, urban practice)

GPs are generally aware of the challenges involved when antibiotic treatment is prescribed, but the use of guidelines is haphazard for this population:

*‘I think we use antibiotics, and I, I, I don’t think there are very many control systems in place, certainly around here. Uh, I think there is an overreliance’* (Male, urban practice)

*‘Right now, it isn’t good. Nitrofurantoin for the past 2 or 3 years has been the number one medicine but it is also going to cause trouble soon enough.’* (Male, urban practice)

*‘Yeah and they give first-line and second-line and obviously you know … Augmentin (co-amoxiclav), ciprofloxacin, all those ones are second or third line agents … you know you wouldn’t be jumping in straight away with them.’* (Female, rural practice)

Challenges in treating male UTIs are also evident in the lack of knowledge of antimicrobial prescribing guidelines by GPs within the study:

‘*I mean doxycyline use has always been standard since I qualified anyway, 10 or 12 years ago ...’* (Male, urban practice)

*‘I would treat them with a first-line antibiotic and not send the urine to the hospital. Because by the time you get the results back, you know whether the antibiotics worked or not*.’ (Male, urban practice)

Particularly in relation to antimicrobial resistance, GPs are aware of this risk, but are not always sure on how this translates to patient care:

*‘I don’t want to be creating quinolone resistance here in the community all on my own.’* (Male urban practice)

‘*I wouldn’t treat the symptoms, I would treat the diagnosis … the treatment depends on the diagnosis.’* (Male, urban practice)

This also reflects the tension between individual patient care and community effects of antimicrobial prescribing.

The difficulty around diagnosing and treating a male UTI is evident in local routine practice, such as the decision to send a urine sample for microbiological assessment.

*‘If I could send a sample to the lab, I would ... But there are situations when you can’t.’* (Male, urban practice)

### GPs’ interactions with their patients in suspected male UTI consultations

When younger males present with symptoms suggestive of a UTI, many GPs first explore the possibility of an STI:

*‘I suppose you’ll always wonder… could it be STI (okay) … that will certainly be the … be the … certainly a man under 35. That’s kind of a … that’s sort of a … arbitrary cut off we have in the community.’* (Male, urban practice)

*‘So in the younger man, we’d already be thinking, I bet any money, this is sexually transmitted disease one way or another.’* (Male, urban practice)

*‘It is distributive of age. In younger males, it would definitely be STI. I mean that’s what you would think about before a UTI.’* (Male, urban practice)

GPs express how older patients often give an elaborative view of all their ailments, which is difficult to treat seriously and make sense of in one single consultation:

*‘ “Can you take a look at this doctor, I also have a bump here doctor, my knee isn’t working, should I get a knee replacement”, ummm and all this has all been included within the same 9 minute consult.’* (Male, urban practice)

While in some cases GPs may experience older patients as confused, this may result in a sense of disbelief towards older patients and their symptoms. Sometimes symptoms do not even adhere to symptoms suggestive of a UTI, in particular in older males with underlying comorbidities such as dementia:

*‘In fact some of them may just present as confused without a single solitary abdominal or urinary or clinical examination finding and that’s the absolute truth.’* (Male, urban practice)

*‘This population coming in, we’re monitoring their dementia.’* (Male, urban practice)

GPs recognise the difficulties experienced by the patient when they contract a UTI; particularly the impact it may have on the patient’s mental health:

*‘… Some of them get very depressed about it.’* (Male, urban practice)

*‘… it can drive men nuts.’* (Male, urban practice)

Due to the complexity of this issue, GPs present empathy for what males are experiencing:

*‘They might be a little bit embarrassed about their symptoms, they might not know what’s going on, or they might feel uncomfortable.’* (Female, urban practice)

In order to improve communication and support better and faster diagnosis, one GP suggested that a brief outline of male UTI and appropriate medication in the form of a shortened booklet would be beneficial to highlight the issues:

*‘Yeah, so, um that would be having specific actually would be helpful actually, even if it was say an academic card or something, or a summary of what’s out there really you don’t have time to check up everything, even when the patients in with you or beforehand. Yeah but that I think would be helpful.’* (Female, urban practice)

## Discussion

### Summary

Overall, three themes were identified which represent the attitudes of GPs when diagnosing and treating male UTIs in urban and rural practices in Ireland. The themes that were detected through thematic analysis were GPs’ experience with diagnosing and treating male UTIs, GPs’ challenges in diagnosing and treating male UTIs, and GPs’ interactions with patients during male UTI consultations. A number of GPs believed that male UTIs were complicated and difficult to diagnose particularly in relation to the age of the patient, with older patients being of greater concern. UTIs in males are considered uncommon compared to female patients. GPs also expressed ambiguity around treatment recommendations and guidelines when prescribing, suggesting a reliance on their clinical knowledge and experience in many cases. Nonetheless, NICE guidelines for the management of UTI symptoms in men have been available since 2011 and were updated in 2015.^18^ They focus on recommendations for initial and specialist assessment, conservative management, and antimicrobial treatment for health practitioners managing UTIs in male patients. Additionally, HSE Antibiotic Prescribing^19^ provides comprehensive treatment recommendations, yet HSE guidelines remain unrevised since 2011 and focus more generally on UTIs in ‘adults’ and children, while frequently referring to females.

### Strengths and limitations

This is a unique study exploring GPs’ attitudes towards diagnosis and treatment of male UTIs, an area in which little research has been done. Through 15 interviews, it became clear that little consensus on diagnosis and treatment exists. All GPs interviewed represented the Irish population of GPs who encounter male UTI consultations: GPs were not included based on clinical expertise in this area or volume of male UTIs seen in any given practice. Interviews were conducted by medical students who had relevant clinical experience to discuss this acute condition with GPs.

A limitation of the study was failure to include an equal number of urban and rural practices; this was an oversight and, when recruiting, was viewed as unnecessary, but in hindsight may have created some clear comparisons in the current findings. The main limitation of the study is that it focused solely on GPs practicing in Ireland who mainly used Irish guidelines when prescribing; a more international focus would show how national guidelines, for example NICE, influence attitudes towards prescribing in different countries.

### Comparison with existing literature

The diagnosis of male UTIs is not straightforward and there is no gold standard.^20^ Diagnosing a male UTI should focus on the individual patient and take the consultation on a case-by-case basis, especially when clinicians are uncertain on the specific diagnosis.^21^ When diagnosing a male UTI, the acute onset of symptoms, lack of systemic symptoms, or history of urological complaints generally suggests that the UTI is uncomplicated,^22^ though additional detection of bacteria can help this diagnosis.^23^ Research shows the main uropathogens for male UTIs are *Escherichia coli*, followed by other enterobacteriaceae and enterococci, all of which are susceptible to nitrofurantoin, the first-line treatment for UTIs in males.^24^ It is clear from the interviews that many GPs find the diagnosis of a male with an (uncomplicated) UTI challenging, but if empirical prescribing is chosen, this should follow national guidelines. Since 2015, NICE guidelines have been updated and provide a comprehensive set of recommendations for GPs in managing urinary tract symptoms in men.^18^ Nonetheless, GPs require a diagnosis of a UTI before they consult with UTI guidelines; it seems that GPs in these interviews are having difficulty confirming a male UTI diagnosis, thus making guideline recommendations more challenging when they are unsure about what they are treating.

There is uncertainty surrounding the diagnosis of male UTIs and appropriate treatment options. This uncertainty also reflects the lack of research on male UTIs. The extrapolation of findings from research on female UTI to males causes problems for the diagnostic and treatment process and adds to the confusion.^25^ Some GPs participating in this study had little experience with males presenting with a UTI, which intensified their feelings of uncertainty. There is a common perception that male UTIs are complicated even though there is no evidence to support this perception.^24^ Age may be considered when diagnosing a male UTI, and this is also reflected in the literature, as the prevalence of UTIs increases rapidly in men aged ≥60 years.^23^ This does not rule out UTI in younger males, however, even though potential STIs have to be checked. Overall, male UTIs are complex and challenging to diagnose.

Adherence to guidelines for treating male UTIs is not optimal as available clinical indications are not formally validated.^25^ The guidelines for treating male UTIs differ greatly, are quite contradictory, and usually differ significantly between regions.^26^ The Infectious Diseases Society of America specifically exclude males from UTI treatment recommendations.^27^

Guidelines for treating male UTIs usually discourage fluoroquinolone use^23^ in light of the spread of AMR as well as known adverse effects, with more recent national guidelines restricting the use of fluoroquinolones due to infrequent, but potentially long-lasting, side effects involving swollen or torn tendons, muscle pain, or weakness and joint pain or inflammation.^28^ Despite this, fluoroquinolones are often prescribed and Kobayashi and colleagues’^29^ study in the US highlighted that almost half of patients were given fluoroquinolones, despite trimethoprim being the first-line recommendation.^25^ The Irish guidelines are clear on prescribing for male uncomplicated UTI, but this was not reflected in the study, probably due to the confusion around diagnosis or perhaps because the majority of GPs interviewed referred to HSE guidelines as their primary source and these guidelines have not been updated since 2011.^19^ The current study also showed the lack of clarity around prescribing for male UTIs and of the participating 15 GPs: 6 suggested they would prescribe a different antibiotic from the guidelines and only 9 GPs suggested either nitrofurantoin or trimethoprim. Perhaps, as Trautner^30^ suggests, a more judicious therapy approach is desired for treating male UTIs. This should involve GPs in Ireland referring to both local-level and national recommendations when prescribing for male UTIs. This study’s findings revealed that only 2 GPs referred to national guidelines. It seems bodies such as NICE, the European Medicines Agency, and Pharmacovigilance Risk Assessment Committee are regularly applying updates to guidelines in this area. For example, NICE guidelines from January 2019 provide an interactive flowchart which gives a clear overview of the assessment, support, and management procedures involved in the diagnosis and treatment of a male UTI.^31^

### Implications for research and practice

These findings show that GPs’ attitudes towards treatment and diagnosis differ greatly and this ambivalence appears common throughout suspected male UTI consultations. More research needs to be undertaken to understand how to improve the diagnosis and treatment of UTI and how to support GPs in making treatment decisions. An emphasis should be placed on how to identify and treat male UTIs in line with current best practice guidelines. A useful suggestion that one female GP made was to create quick tip guideline leaflets on male UTI diagnosis and treatment options, that GPs could keep on their desk and refer to if uncertain. This GP was confident this would be a lot more efficient than having to search for guidelines and recommendations during the consultation*.*

Few studies have considered the best approaches to distinguishing clinical criteria for UTI in males. Moreover there are few randomised control trials available which focus on male UTIs. One review suggests a large prospective observational study enrolling males with microbiologically confirmed UTIs which would minimise diagnosis and treatment based on symptom presentation alone.^32^ However, due to the wide variation in diagnosis and treatment approaches, such a study would require a large sample size resulting in challenges concerning cost, recruitment, and retention.^23,32^ Given the more recent recommendations restricting the use of quinolone and fluoroquinolone treatment for acute infections,^28^ an international randomised clinical trial comparing first-line treatment with fluoroquinolone (which is accepted in most countries as second-line or treatment for complicated UTIs) would be efficient and appropriate, followed by or embedded with a trial considering treatment duration.
